# Correction: Full-field optical coherence tomography for the diagnosis of giant cell arteritis

**DOI:** 10.1371/journal.pone.0296315

**Published:** 2023-12-19

**Authors:** Thomas Maldiney, Hélène Greigert, Laurent Martin, Emilie Benoit, Catherine Creuzot-Garcher, Pierre-Henry Gabrielle, Jean-Marie Chassot, Claude Boccara, Daniel Balvay, Bertrand Tavitian, Olivier Clément, Sylvain Audia, Bernard Bonnotte, Maxime Samson

The legends for Figs 1–4 are missing from the article. The captions have been provided here:

**Fig 1. TAB architecture imaging.** Arterial wall (A, D), *vasa vasorum* (B, E), and red blood cells (C, F) imaging with FF-OCT (A to C) and conventional histology following HES staining (D to F). Scale bars represent 50 µm. Black arrows show the IEL. White arrows show arterial thrombosis. Asterisks mark red blood cells. Legend: a, adventitia; i, intima; m, media.

**Fig 2. Qualitative imaging of TAB specimens.** Comparison of FF-OCT (A, C, E) and conventional histology (B, D, F) imaging. A and B correspond to niTABs (n = 9), C and D to ihTABs (n = 3), E and F to gcaTABs (n = 4). Scale bars represent 100 µm. Black arrows show the IEL. White arrows show rupture of the circular symmetry and mononuclear infiltrate. Legend: a, adventitia; i, intima; m, media.

**Fig 3. Image analysis of TAB sections.** ihTAB1 FF-OCT translational section (A). ImageJ graphical plot of ihTAB1 FF-OCT translational section (B). Orientation maps after Gabor filtering of ihTAB1 FF-OCT translational section (C). gcaTAB3 FF-OCT translational section (D). ImageJ graphical plot of gcaTAB3 FF-OCT translational section (E). Orientation maps after Gabor filtering of gcaTAB3 FF-OCT translational section (F).

**Fig 4. Quantitative analysis of TAB sections.** FF-OCT and conventional histology (HI) cross-sectional match for the visualization of the temporal artery wall (A). Comparison of FF-OCT and HI-based intima-to-media ratios for both healthy and GCA-positive TAB sections (B). Correlation curves between FF-OCT and HI for the measurement of intima (C) and media (D) thickness. Scale bars represent 100 µm. Legend: a, adventitia; i, intima; m, media.
