# Supplementary figures and images for: Full-field optical coherence tomography for the diagnosis of giant cell arteritis
Source: PLoS One. 2020 Aug 31;15(8):e0234165. doi: 10.1371/journal.pone.0234165 (PMC7458309; doi:10.1371/journal.pone.0234165)

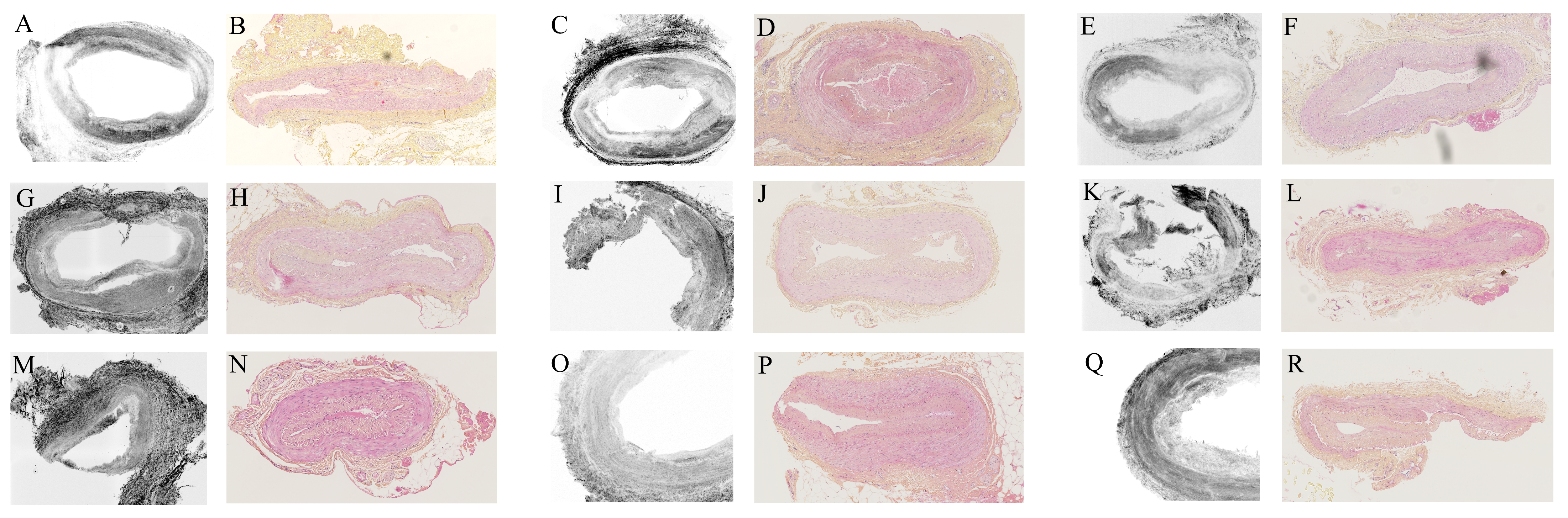

Supplement: S1 Fig — Comparison of FF-OCT (A, C, E, G, I, K, M, O, Q) and conventional histology (B, D, F, H, J, L, N, P, R) imaging. A and B correspond to niTAB1, C and D to niTAB2, E and F to niTAB3, G and H to niTAB4, I and J to niTAB5, K and L to niTAB5, M and N to niTAB7, O and P to niTAB8, Q and R to niTAB9. (TIF) [file pone.0234165.s001.tif]

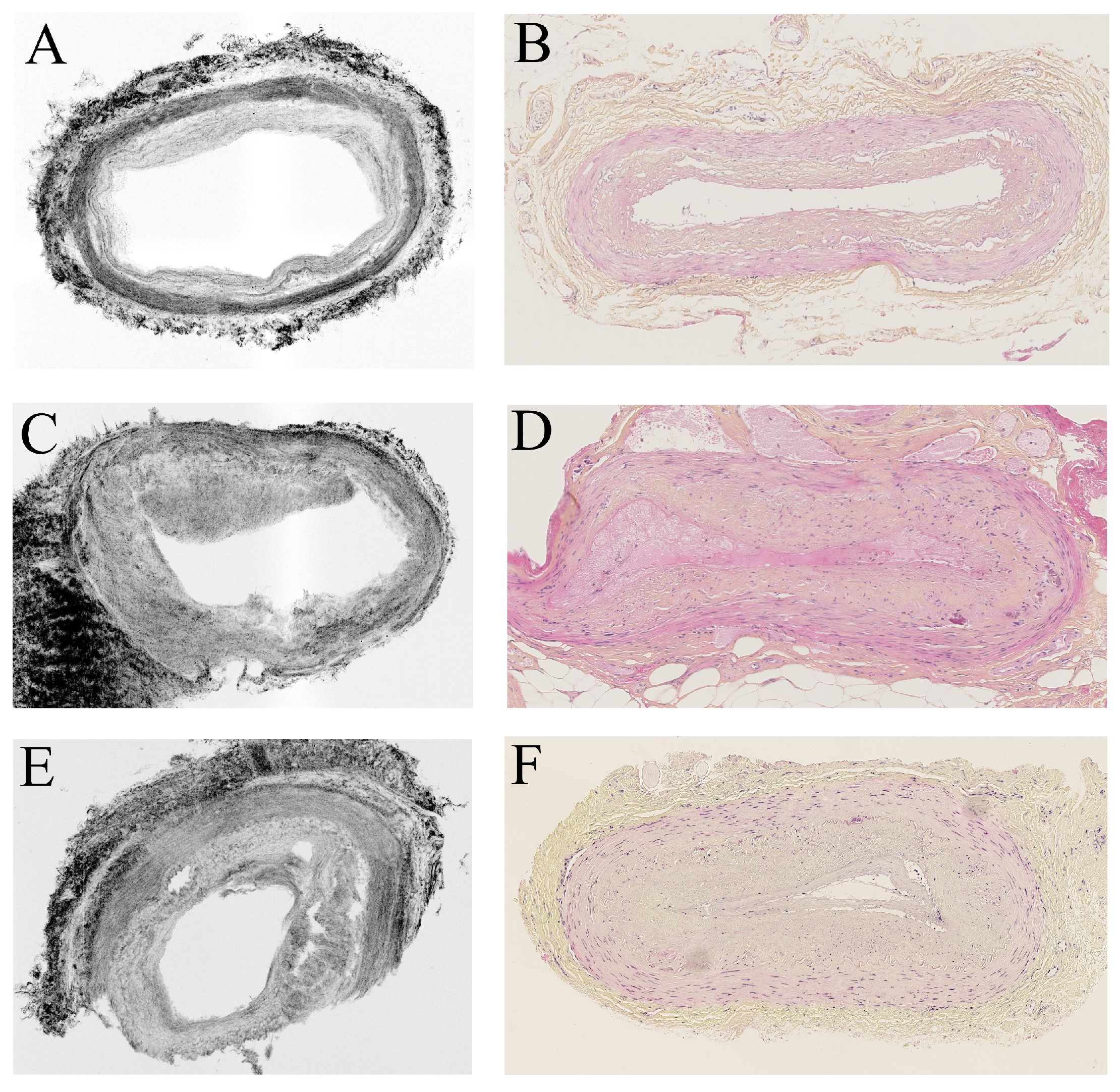

Supplement: S2 Fig — Comparison of FF-OCT (A, C, E) and conventional histology (B, D, F) imaging. A and B correspond to the ihTAB1, C and D to ihTAB2, E and F to ihTAB3. (TIF) [file pone.0234165.s002.tif]

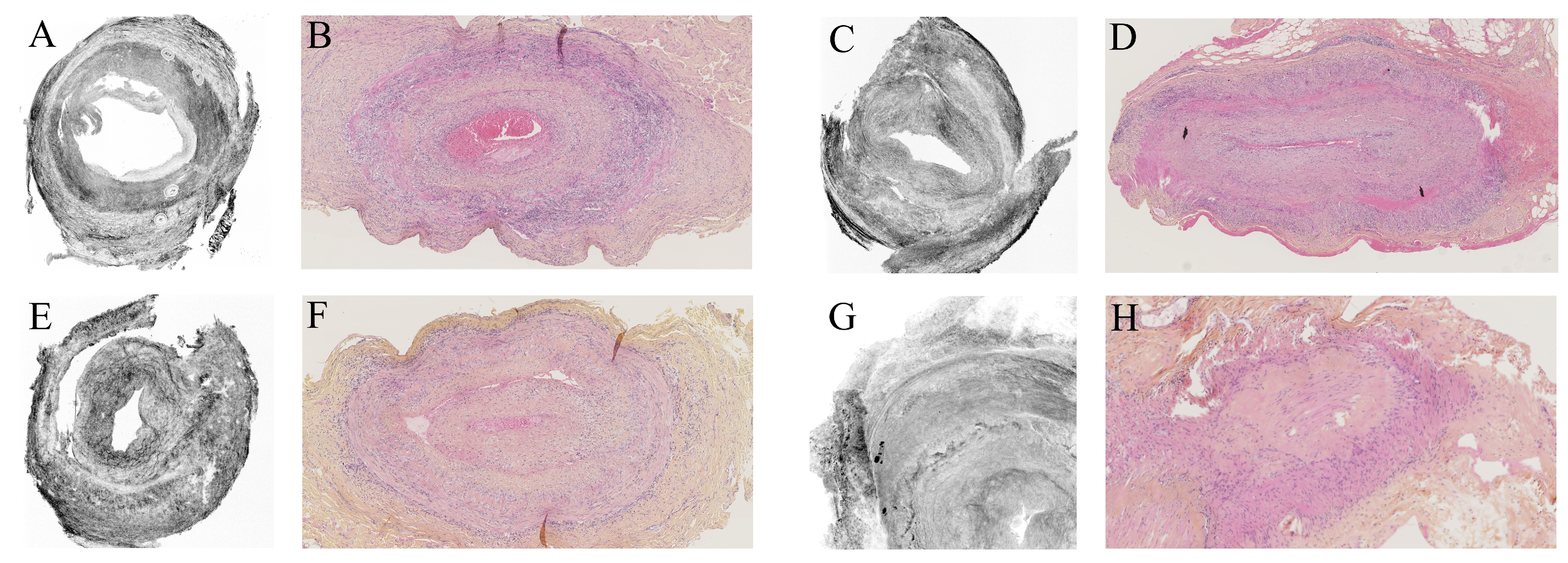

Supplement: S3 Fig — Comparison of FF-OCT (A, C, E, G) and conventional histology (B, D, F, H) imaging. A and B correspond to gcaTAB1, C and D to gcaTAB2, E and F to gcaTAB3, G and H to gcaTAB4. (TIF) [file pone.0234165.s003.tif]
